# β-Amyloid oligomers in aging and Alzheimer’s disease

**DOI:** 10.3389/fnagi.2013.00028

**Published:** 2013-07-04

**Authors:** Kathleen R. Zahs, Karen H. Ashe

**Affiliations:** ^1^N. Bud Grossman Center for Memory Research and Care, University of MinnesotaMinneapolis, MN, USA; ^2^Department of Neurology, University of MinnesotaMinneapolis, MN, USA; ^3^Department of Neuroscience, University of MinnesotaMinneapolis, MN, USA; ^4^Geriatric Research Education Clinical Center, VA Medical CenterMinneapolis, MN, USA

**Keywords:** β-amyloid, oligomer, Alzheimer’s disease, Aβ*56, preclinical Alzheimer’s disease

## Abstract

Alzheimer’s disease (AD) is a fatal neurodegenerative disorder, and the most common cause of dementia in the elderly. The cause of AD is not known, but genetic evidence strongly supports the hypothesis that pathological aggregation of the β-amyloid protein (Aβ) triggers the disease process. AD has a long preclinical phase, lasting a decade or more. It is during this preclinical phase, before the irreversible neuron loss that characterizes the dementia phase of the disease, that therapies are most likely to be effective. If we are to block AD during the preclinical phase, we must identify the Aβ species that are present before there are overt symptoms and that are associated with downstream markers of pathology. A specific soluble Aβ assembly, the putative dodecamer “Aβ*56,” is present in the brains and cerebrospinal fluid of cognitively intact individuals and correlates with markers of synaptic dysfunction and neuronal injury. This assembly also correlates with memory dysfunction in multiple lines of transgenic mice that model the preclinical phase of AD. We suggest that Aβ*56 has a critical role during the earliest phase of AD and might serve as a molecular trigger of the disease.

## INTRODUCTION

Alzheimer’s disease (AD) is the most common cause of dementia in the elderly. Age is the greatest risk factor for AD; an estimated 13% of Americans over age 65 suffer from AD, while that number increases to 45% for those aged 85 and older (Alzheimer’s Association, 2012). The disease is defined by characteristic lesions in the brain – amyloid plaques composed of aggregated β-amyloid protein (Aβ), neurofibrillary tangles composed of hyperphosphorylated tau protein, and widespread synapse and neuron loss. In addition to these lesions, oxidative stress, neuroinflammation, and aberrant cell-cycle re-entry are among the pathological features observed in AD brains (Mondragon-Rodriguez et al., 2010). The cause of AD is not known, and controversy persists over which abnormalities initiate the disease process, which are responses that potentiate neurodegeneration, and even whether some of these abnormalities represent protective responses of the brain (Mondragon-Rodriguez et al., 2010).

The identification of genes that influence susceptibility to AD has led to insights into potential disease mechanisms. Although most cases of AD arise sporadically, in rare cases the disease is caused by autosomal-dominant mutations. All known mutations that cause familial autosomal-dominant AD increase the production of Aβ or its propensity to aggregate (reviewed in Ashe and Zahs, 2010). Conversely, a mutation in the amyloid precursor protein (APP) that results in decreased production of Aβ protects against sporadic AD (Jonsson et al., 2012). The best established genetic risk factor for sporadic AD is the ε4 allele of the APOE (apolipoprotein E) gene – compared to individuals with the most common genotype (ε3/ε3), individuals with one copy of the ε4 allele are three times more likely to develop AD, while those with two copies of the ε4 allele are 10–15 times more likely to develop AD (Corder et al., 1993). While the exact role that APOE plays in AD is not known, animal studies have shown that the rate of Aβ clearance from the brain is differentially regulated by the various isoforms of APOE (Castellano et al., 2011). Thus, the genetic evidence points to some form of Aβ – likely a pathological aggregate – as the molecular trigger for AD.

Mutations in tau, on the other hand, do not lead to AD, but to a different neurodegenerative disorder, frontotemporal dementia (FTD; Hutton et al., 1998). Transgenic mice expressing human tau with FTD-linked mutations exhibit pronounced neurodegeneration (Lewis et al., 2000; Ramsden et al., 2005; Santacruz et al., 2005; Yoshiyama et al., 2007). Notably, many of the post-translational modifications in tau that are promoted by FTD-linked mutations are also seen in AD brains (Ramsden et al., 2005; Yoshiyama et al., 2007). Furthermore, studies in animal models have shown that Aβ potentiates tau pathology (Gotz et al., 2001; Lewis et al., 2001; Oddo et al., 2004; Bolmont et al., 2007). Finally, tau is required for the expression of Aβ-induced neurological abnormalities in transgenic mice (Roberson et al., 2007).

Taken together, the evidence cited above provides strong support for the Amyloid Cascade Hypothesis, which posits that pathogenic forms of Aβ trigger a cascade that leads to the formation of toxic tau species and culminates in neuron death and dementia. While the Amyloid Cascade Hypothesis is arguably the primary hypothesis driving AD research today, it has its detractors (Castellani and Smith, 2011). Opponents of the hypothesis point to the failure in clinical trials of drugs that target amyloid; while supporters contend that treatments were administered too late to be effective – at a time when neurodegeneration had become independent of the toxic Aβ species that initiated the disease process. To truly test the Amyloid Cascade Hypothesis will require intervening very early in the disease process, likely before symptoms are apparent, *and* targeting the correct form of Aβ. The identification of the pathogenic form(s) of Aβ is the focus of this *Perspective*.

## Aβ OLIGOMERS IN THE HUMAN CENTRAL NERVOUS SYSTEM

One of the most profound questions for AD researchers today is which Aβ species triggers the amyloid cascade. In addition to amyloid plaques, which contain precipitates of fibrillar Aβ, several types of soluble Aβ assemblies (“oligomers”) have been described in the brains of AD patients and transgenic mouse models of AD. Studies conducted over the past decade indicate that oligomers, rather than fibrillar Aβ, are the predominant bioactive forms; synthetic and naturally derived Aβ oligomers harm cultured neurons and impair synaptic function and memory through a broad range of mechanisms (reviewed in Ashe and Zahs, 2010; Koffie et al., 2011). Identifying the oligomer that initiates the amyloid cascade is not just of academic interest – this knowledge is critical for the development of strategies to prevent AD.

Alzheimer’s disease has a long preclinical phase, lasting a decade or more (Sperling et al., 2011; Bateman et al., 2012; Villemagne et al., 2013). During this preclinical phase, people appear cognitively intact (i.e., they score within the normal range on neuropsychiatric tests), but evidence of neurological disease can be seen through cerebrospinal fluid (CSF) or brain imaging analyses. It is during this preclinical phase, before the irreversible neuron loss that characterizes the dementia phase of the disease, that therapies are most likely to be effective. If we are to block AD during the preclinical phase, we must identify the Aβ species that are present before there are overt symptoms and are associated with downstream markers of synaptic or neuronal pathology.

Two studies published this year measured levels of specific Aβ oligomers in the brains and CSF of human subjects – both reported that the putative dodecamer Aβ*56 (Lesné et al., 2006) correlated with markers of neuronal dysfunction or injury in cognitively normal subjects. Lesné et al. (2013) measured brain Aβ dimers, Aβ trimers, and Aβ*56 in 140 autopsy specimens from subjects spanning 10 decades of age. In subjects who were cognitively normal at the time of death, Aβ*56, but not other Aβ oligomers, correlated negatively with the post-synaptic markers drebrin and Fyn kinase and positively with pathological conformers of tau (Lesné et al., 2013). In the first study to measure levels of specific Aβ oligomers in the CSF of cognitively normal older adults, Handoko et al. (2013) found that Aβ*56 was elevated in individuals at risk for developing AD and correlated strongly with levels of total tau and tau phosphorylated at threonine-181, putative markers of neuronal injury. Interestingly, the levels of Aβ*56 in the brain rose significantly in late middle age (Lesné et al., 2013) – considering the long duration of the preclinical phase of AD, this is the time when one would expect the emergence of species that trigger the amyloid cascade.

It should be noted that other Aβ assemblies, in addition to those studied by Lesné et al. (2013) and Handoko et al. (2013), have been described in the brains of AD patients (Noguchi et al., 2009; Lasagna-Reeves et al., 2011). Whether these species exist in the preclinical phase of AD and whether they are associated with downstream markers of pathology are yet to be determined.

## Aβ OLIGOMERS IN TRANSGENIC MOUSE MODELS OF ALZHEIMER’S DISEASE

Studies in animal models have provided further insights into the pathophysiological roles of specific oligomers. Among the Aβ species detected in the brains of AD patients or transgenic mouse models of AD, only two have been shown to induce neural dysfunction when isolated and injected into the brains of healthy host animals: Aβ*56 (Lesné et al., 2006; Reed et al., 2009) and Aβ dimers (Klyubin et al., 2008; Shankar et al., 2008; Barry et al., 2011; our unpublished observations). Additionally, Aβ*56 correlates with memory deficits in three distinct lines of APP transgenic mice (Lesné et al., 2006, Lesné et al., 2008; Billings et al., 2007; Cheng et al., 2007). (It might at first seem inconsistent that Aβ*56 is elevated in humans who are considered “cognitively intact,” but that it is associated with cognitive dysfunction in rodents. However, it is very possible that cognitive decline would be revealed in these human subjects if they were tested longitudinally using sensitive neuropsychiatric instruments designed for detecting cognitive changes in clinically unimpaired individuals; Rogers, 2013.) Until recently, it has been difficult to assess the effects of Aβ dimers *in situ* (naturally located in the brain in which they were produced), due to a lack of animal models that generated dimers in the absence of Aβ*56. Our laboratory recently created a novel transgenic mouse that generates abundant plaques and Aβ dimers but negligible levels of other Aβ oligomers. These mice remain cognitively normal, even at advanced ages. This result was surprising in view of the *ex situ* studies demonstrating the toxicity of Aβ dimers, cited above. To explain these paradoxical findings, we hypothesized that dimers *in situ* are compartmentalized in a way that limits their toxicity. Biochemical studies have shown an intimate relationship between dimers and plaques (Roher et al., 1996; Shankar et al., 2008). Using laser microdissection followed by immunoblotting, we found that dimers *in situ* are confined to the immediate vicinity of plaques, while Aβ*56 is diffusely distributed throughout the brain parenchyma (Liu et al., 2011). We concluded that despite their potent neurotoxicity when dispersed into cell cultures (Roher et al., 1996; Jin et al., 2011) or into the brains of experimental hosts (Klyubin et al., 2008; Shankar et al., 2008; Barry et al., 2011), dimers *in situ* exert few or no large-scale effects on brain function.

This conclusion highlights the necessity of studying candidate pathogenic molecules/processes *in situ* in order to gain a more genuine understanding of their effects in the brain. While *in vitro* studies can provide valuable information about cellular mechanisms of action, cell culture conditions do not mimic the spatiotemporal expression of pathogenic molecules or the complex intercellular interactions that occur *in vivo*. Similarly, exogenous administration of candidate pathogenic molecules can show the toxic potential of these molecules, but not necessarily their effects on neurological function when naturally produced and localized in the brain. This caution does not apply only to studies of Aβ – a recent essay in this series argued that extrapolating the results of *in vitro* studies to the situation *in vivo* has resulted in a gross misunderstanding of the role of microglia in chronic brain disease (Streit, 2010).

## DUAL-PATHWAY MODEL FOR Aβ TOXICITY IN THE BRAIN

**FIGURE 1:**
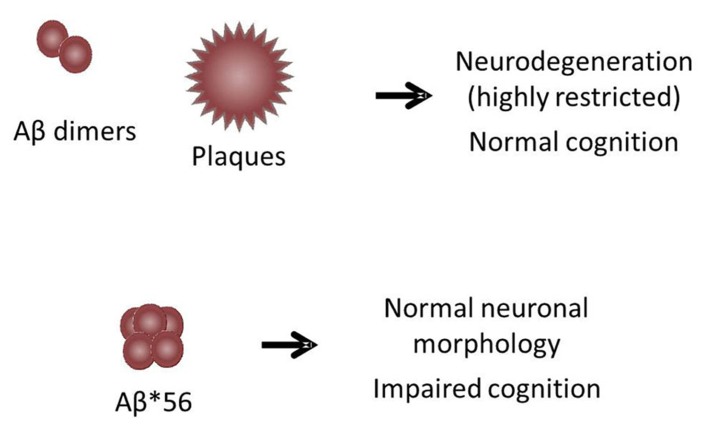
** Dual-pathway model for Aβ toxicity in the brain.** The model illustrates two ways in which Aβ oligomers can be toxic: (1) Aβ*56, which is diffusely distributed throughout the brain parenchyma, induces widespread synaptic dysfunction, leading to pathological processing of tau and subsequent neurodegeneration over a wide area. (2) Aβ dimers, which are tightly sequestered around plaques, induce neurodegeneration only in the immediate vicinity of plaques. The area influenced by dimers is not large enough to impair cognitive function in animals with plaque loads that approximate those found in humans.

Based on the findings discussed above, we suggest that there are at least two ways in which Aβ oligomers can be toxic, as illustrated in the model in **Figure 1**. In this model, Aβ*56 is diffusely distributed throughout the brain parenchyma and induces widespread synaptic dysfunction, which eventually leads to pathological processing of tau and subsequent neurodegeneration over a wide area. Aβ dimers are potently neurotoxic and induce neurodegeneration, but the tight sequestration of dimers to the immediate vicinity of plaques spatially restricts their influence. This model has important implications for the development of strategies to prevent AD. We hypothesize that, in order to prevent or delay the onset of dementia, anti-amyloid therapies must not only be administered early in the presymptomatic phase of the disease, but must target Aβ*56. According to the model, strategies that target Aβ dimers and and/or fibrils might correct plaque-associated neurodegenerative changes if administered early enough, but will have little impact on the development of the widespread neuronal dysfunction and degeneration that characterize AD.

## THE ROLE OF Aβ IN AGE-RELATED COGNITIVE DECLINE

As mentioned in the Introduction, age is the greatest risk factor for AD. It is not known whether AD falls at the extreme end of a continuum of normal age-related cognitive decline or whether it represents a distinct pathological process. The same mutation in APP that protects against AD also protects against age-related cognitive decline (Jonsson et al., 2012), suggesting that Aβ has a role in both conditions. Our laboratory previously reported that expression of transgenic APP accelerated the appearance of age-related neural dysfunction in mice (Hsiao et al., 1995). These studies occurred prior to the time that we began characterizing soluble oligomers in the brains of our mice, so we do not know which Aβ species were involved, although notably these mice did not generate plaques. Observations in senescence-accelerated prone mice (SAMP) are consistent with the hypothesis that Aβ is involved in “normal” brain aging; these mice develop deficits in learning and memory at young ages relative to the parent strain and have elevated levels of endogenous mouse APP and Aβ, but no plaques (Okuma and Nomura, 1998). Administration of antibodies against Aβ or anti-sense nucleotides that lower APP mRNA ameliorates cognitive deficits in these mice (Kumar et al., 2000; Morley et al., 2000; Banks et al., 2001).

## FUTURE DIRECTIONS AND CONCLUSIONS

The data available thus far strongly suggest that Aβ*56 is critically involved in the earliest stages of AD. However, longitudinal studies are required to determine whether “cognitively normal” individuals with elevated levels of Aβ*56 show accelerated rates of cognitive decline or are indeed more likely to develop AD than people with low levels of Aβ*56. These studies might also shed light on the question of whether age-related cognitive decline and AD share similar mechanisms and are indeed on a continuum of brain aging. Such studies are still only correlative. To really test the hypothesis that Aβ*56 is necessary to trigger the amyloid cascade, we must determine whether selectively decreasing the levels of Aβ*56, or interfering with its interactions with its cellular targets, reduces the risk of symptomatic AD. Such studies await the development of reagents that selectively target specific Aβ oligomers.

Three well publicized AD prevention trials are scheduled to begin this year (2013): the Alzheimer’s Prevention Initiative (API) and Dominantly Inherited Alzheimer’s Network (DIAN) trials will test anti-amyloid therapies in asymptomatic members of families with autosomal-dominant AD, while the Anti-amyloid Treatment in Asymptomatic Alzheimer’s Disease (A4) trial will examine the effects of therapy on individuals at increased risk of sporadic AD, enrolling elderly people with who have amyloid-positive PET scans. All of these trials will test the effects of monoclonal antibodies directed against Aβ, but it is not known whether any of the antibodies to be tested in these critical prevention trials target Aβ*56. The API, DIAN, and A4 trials are considered by many to be the first true tests of the Amyloid Cascade Hypothesis. Should these trials fail because they did not target the relevant Aβ species, the result could be the premature rejection of the amyloid cascade hypothesis and the abandonment of drug development programs that target Aβ. Continued failures of clinical trials will further undermine public confidence in biomedical research and, most importantly, delay the implementation of therapies that will lessen the impending public health crisis that is AD. Future research clarifying *which* forms of Aβ to target and *where* the targets are located will be critical for the development of mechanism-based therapies for AD.

## Conflict of Interest Statement

The authors declare that the research was conducted in the absence of any commercial or financial relationships that could be construed as a potential conflict of interest.
